# A Method of Utilizing Atmospheric Pressure for the Retention of Lower Plates

**Published:** 1910-03-15

**Authors:** D. H. Young

**Affiliations:** Attica, N. Y.


### A METHOD OF UTILIZING ATMOSPHERIC PRESSURE FOR THE RETENTION OF LOWER PLATES.

BY D. H. YOUNG, D.D.S., ATTICA, N. Y.

In order to secure atmospheric pressure on the lower plate we must proceed by a method that has been formulated with the idea of taking into consideration the various conditions with which we meet in different mandibles, for if we can make a plate for the lower jaw that will exclude the air from under it, there is no reason why atmospheric pressure will not act as well there as with the upper jaw.

I use, for taking impressions of these cases, Perfection Compound. This material is warmed to a consistency at which it will adapt itself readily to the shape of the mouth.

It is then placed upon the tray, put in the mouth, and pressed down almost as far as required. Then wait for a minute or two until it hardens slightly. During this interval of time I have my assistant direct a current of cold air into the mouth from a rotary fan in the dental engine, which procedure chills the outer layer of the compound and prevents it from flowing away from the jaw when pressure is put upon it. While the layer of impression material that is in contact with the tissues is still warm and plastic, the tray is pressed down just a little farther, using from five to ten pounds pressure, and is then held steadily—with about half the force used in pressing it down the second time—until it is quite hard. The tray is then removed and the cast is poured.

In order that we may know why we proceed in this manner, let us go over the taking of this impression again. In the first place, when the material is put in the mouth and pressed down, we get an ordinary impression. After that is allowed to stand for a short time, and the outside is chilled and pressed down a second time, the impression is changed from the ordinary one, but in what way? When pressing down on the tray the second time the hard ridge of the alveolus cuts just a little deeper into the impression material and remains practically unchanged, while the soft tissues down on the sides of the gums about where the border of the plate reaches are compressed slightly. When the plate is made on the cast from this impression, and put into the mouth, the margins of the plate are sealed all the way around, and this converts the whole lower surface of the plate into one great suction pad—if we may use that term.

One of the most difficult parts of the operation is the trimming of the plate to just that line that will best seal its borders, for if it be too long it will give the muscles an opportunity to displace it. If too short, it may allow air to pass in, and thus defeat our purpose. It is self-evident that the upper plate is retained by air-pressure against gravity, while the lower plate adds its own weight to that of the atmospheric pressure; and so, compared with the upper, the lower has twice its own weight to add to the atmospheric pressure, thus showing that it is a better subject to be retained by such pressure than is the upper plate.—*Summary*, May, 1909.

